# The Cross Talk between TbTim50 and PIP39, Two Aspartate-Based Protein Phosphatases, Maintains Cellular Homeostasis in Trypanosoma brucei

**DOI:** 10.1128/mSphere.00353-19

**Published:** 2019-08-07

**Authors:** Anuj Tripathi, Ujjal K. Singha, Victor Paromov, Salisha Hill, Siddharth Pratap, Kristie Rose, Minu Chaudhuri

**Affiliations:** a Department of Microbiology, Immunology, and Physiology, Meharry Medical College, Nashville, Tennessee, USA; b Department of Biochemistry, Vanderbilt University, Nashville, Tennessee, USA; University of Texas Southwestern

**Keywords:** AMPK, PIP39, Tim50, *Trypanosoma brucei*, stress tolerance

## Abstract

Trypanosoma brucei, the infectious agent of a deadly disease known as African trypanosomiasis, undergoes various stresses during its digenetic life cycle. We previously showed that downregulation of T. brucei mitochondrial inner membrane protein translocase 50 (TbTim50), an aspartate-based protein phosphatase and a component of the translocase of the mitochondrial inner membrane (TIM), increased the tolerance of procyclic cells to oxidative stress. Using comparative proteomics analysis and further validating the proteomics results by immunoblotting, here we discovered that TbTim50 downregulation caused an approximately 5-fold increase in the levels of PIP39, which is also an aspartate-based protein phosphatase and is primarily localized in glycosomes. A moderate upregulation of a number of glycosomal enzymes was also noticed due to TbTim50 knockdown. We found that the rate of mitochondrial ATP production by oxidative phosphorylation decreased and that substrate-level phosphorylation increased due to depletion of TbTim50. These results were correlated with relative increases in the levels of trypanosome alternative oxidase and hexokinase and a reduced-growth phenotype in low-glucose medium. The levels and activity of the mitochondrial superoxide dismutase and glutaredoxin levels were increased due to TbTim50 knockdown. Furthermore, we show that TbTim50 downregulation increased the cellular AMP/ATP ratio, and as a consequence, phosphorylation of AMP-activated protein kinase (AMPK) was increased. Knocking down both TbTim50 and TbPIP39 reduced PIP39 levels as well as AMPK phosphorylation and reduced T. brucei tolerance to oxidative stress. These results suggest that TbTim50 and PIP39, two protein phosphatases in mitochondria and glycosomes, respectively, cross talk via the AMPK pathway to maintain cellular homeostasis in the procyclic form of T. brucei.

**IMPORTANCE** Trypanosoma brucei, the infectious agent of African trypanosomiasis, must adapt to strikingly different host environments during its digenetic life cycle. Developmental regulation of mitochondrial activities is an essential part of these processes. We have shown previously that mitochondrial inner membrane protein translocase 50 in T. brucei (TbTim50) possesses a dually specific phosphatase activity and plays a role in the cellular stress response pathway. Using proteomics analysis, here we have elucidated a novel connection between TbTim50 and a protein phosphatase of the same family, PIP39, which is also a differentiation-related protein localized in glycosomes. We found that these two protein phosphatases cross talk via the AMPK pathway and modulate cellular metabolic activities under stress. Together, our results indicate the importance of a TbTim50 and PIP39 cascade for communication between mitochondria and other cellular parts in regulation of cell homeostasis in T. brucei.

## Abstract (precis)

Trypanosoma brucei, the infectious agent of African trypanosomiasis, must adapt to strikingly different host environments during its digenetic life cycle. Developmental regulation of mitochondrial activities is an essential part of these processes. We have shown previously that mitochondrial inner membrane protein translocase 50 in T. brucei (TbTim50) possesses a dually specific phosphatase activity and plays a role in the cellular stress response pathway. Using proteomics analysis, here we have elucidated a novel connection between TbTim50 and a protein phosphatase of the same family, PIP39, which is also a differentiation-related protein localized in glycosomes. We found that these two protein phosphatases cross talk via the AMPK pathway and modulate cellular metabolic activities under stress. Together, our results indicate the importance of a TbTim50 and PIP39 cascade for communication between mitochondria and other cellular parts in regulation of cell homeostasis in T. brucei.

## INTRODUCTION

Mitochondria play crucial roles in many cellular activities, which include energy production, signal transduction, calcium homeostasis, innate immunity, and apoptosis (1–3). To perform these functions, mitochondria require about a thousand proteins (4). A vast majority (99%) of these proteins are encoded in the nuclear genome and need to be imported into the organelle via translocases of the mitochondrial outer membrane (TOM) and inner membrane (TIM) (5, 6). TOM and TIMs are multisubunit protein complexes. Virtually all nucleus-encoded mitochondrial proteins require the TOM complex to enter through the mitochondrial outer membrane (MOM) (7). Once they cross the TOM complex, precursor proteins are translocated via either the TIM23 or TIM22 complex, depending on the type of targeting signals that they have and also on their destination, such as the mitochondrial inner membrane (MIM), intermembrane space (IMS), or matrix (5, 6).

The TIM23 complex, also known as preprotein translocase, consists of 3 major proteins, Tim23, Tim17, and Tim50 (8, 9). Tim23 and Tim17 form the import channel of the TIM23 complex, and Tim50 acts as the receptor for preproteins. In contrast to Tim23 and Tim17, which are primarily embedded in the MIM, Tim50 possesses a large hydrophilic domain at the C-terminal half of the protein that is exposed in the IMS. In yeast, the N-terminal region of Tim23 is also exposed in the IMS and extended into the MOM to make contact with the TOM complex (10, 11). The IMS-exposed regions of Tim50 and Tim23 interact with each other and create a gate for the TIM23 protein translocation channel (12). The C-terminal domain (CTD) of Tim50 possesses a phosphatase motif, which belongs to the phosphatase family that dephosphorylates the C-terminal repeat region of the RNA polymerase II large subunit (13). These are aspartate-based protein phosphatases that possess a signature motif, DXDX(T/V), at the catalytic site and are collectively known as CTD phosphatases (14). Fungal Tim50 lacks a perfect motif and does not have phosphatase activity (5). On the other hand, Tim50 in other eukaryotes, like mammals, insects, plants, and fish, possesses this motif, and human Tim50 showed phosphatase activity (15–17). It has been reported that besides mitochondrial protein import, Tim50 is involved in various cellular functions in these organisms, such as steroidogenesis in gonadal tissues (18), protection of cardiac cells from cardiac hypertrophy (19), apoptosis and developmental regulation in *Drosophila* and zebrafish (15, 16), growth and proliferation in plants (17), and chemoresistance in cancer cells (20). It is known that Tim50 maintains the permeability barrier of the MIM; therefore, over- or underexpression of Tim50 hampers mitochondrial function by hyperpolarization or depolarization of the mitochondrial membrane, respectively (12). However, a further mechanism of Tim50’s action in other cellular functions is not clear.

Trypanosoma brucei, a unicellular parasitic protozoon, causes a vector-borne disease in human and domestic animals known as African trypanosomiasis, which is often fatal if not treated (21). During its digenetic life cycle, T. brucei undergoes differentiation to multiple stages to adapt within the insect vector as the procyclic form and in the mammalian host as the bloodstream form (21). Stage differentiation in T. brucei is associated with changes in morphology, the metabolic pattern, surface protein expression, and mitochondrial activities (22, 23) that require a coordinated regulation of gene expression, which in trypanosomatids primarily occurs posttranscriptionally (24).

T. brucei possesses a single reticular mitochondrion that spans the entire cell body (25). Depending on nutritional availability and other environmental changes, T. brucei alters its mitochondrial activities dramatically (26). Similar to other eukaryotes, the single mitochondrion in T. brucei encodes only a limited number of proteins. Therefore, about a thousand nucleus-encoded proteins are imported into the organelle from the cytosol (27, 28). In order to perform this task, T. brucei owns dedicated mitochondrial protein import machinery; however, many of the components of the protein translocase complexes are uniquely divergent in trypanosomes (28–32). T. brucei possesses a single TIM complex (30, 31). TbTim17, the major component of the TbTIM, is apparently the most homologous subunit among all other TbTims (33). T. brucei also possesses a homolog of Tim50, which has been shown to be associated with TbTim17 and involved in mitochondrial protein import (34).

Previously, we characterized TbTim50 and showed that it possesses the signature ^242^DXDXT^246^ motif and also has phosphatase activity (34). Although the T. brucei genome encodes several other aspartate-based protein phosphatases besides TbTim50, only the protein tyrosine phosphatase 1 (PTP1)-interacting protein (PIP39) with a similar motif has been functionally characterized (35). PIP39 is localized in the glycosome as well as in the cytosol and acts as the substrate for PTP1 (35). Both PTP1 and PIP39 are differentiation-related protein phosphatases (35, 36). PIP39 expression and activity are upregulated in the nonreplicating stumpy bloodstream form, which is predominantly found at the peak parasitemic condition in the mammalian bloodstream. The PIP39 protein level is highest in the procyclic form (35, 37). It has been shown that PIP39 is localized in the glycosome, a peroxisome-like organelle that harbors most of the enzymes for glycolytic, ether-lipid biosynthesis, and pentose phosphate metabolic pathways, and it is critical for the transition from the stumpy (ST) bloodstream to the procyclic form of T. brucei (35). However, its function in the procyclic form has not been further investigated.

We have previously shown that depletion of TbTim50 reduced mitochondrial membrane potential and increased the levels of reactive oxygen species (ROS) moderately (38). As a consequence, T. brucei with a reduced level of TbTim50 became more tolerant and stayed at the early apoptosis-like stage upon external addition of H_2_O_2_, indicating that Tim50 may play roles to maintain cellular homeostasis (38). Here, we extend our studies and show that downregulation of TbTim50 increased the cellular AMP/ATP ratio, which activates AMP-activated protein kinase (AMPK) and causes a rearrangement of the cellular proteomes, including an upregulation of PIP39, which is required for tolerance to oxidative stress.

## RESULTS

### TbTim50 knockdown increases the levels of PIP39.

To understand the overall effect of changes in TbTim50 levels on cellular proteomes, we performed multidimensional protein identification technology (MudPIT) analysis with label-free relative quantitation of protein levels. Equal amounts of proteins of the crude mitochondrial lysates from parental control and TbTim50 knockdown (KD) T. brucei cells were digested with trypsin and analyzed by nano-liquid chromatography-tandem mass spectrometry (LC-MS/MS). Mass spectra were searched against the combined NCBI database. After applying protein filters (false discovery rate [FDR] of <1% and at least two unique peptides), over 1,200 T. brucei proteins were identified with significant scores (see Data Set S1 in the supplemental material). Data obtained from two independent biological replicates were used to estimate statistical significance. Following statistical analysis, we found 131 differentially expressed proteins with *P* values of <0.05. Among these, 15 proteins were upregulated (1.5- to 10-fold) (Table 1) and 60 were downregulated (0.14- to 0.5-fold) in TbTim50 knockdown compared to wild-type cells (Table 2). Gene ontology analysis revealed a single protein, PIP39, that was consistently upregulated by T. brucei TbTim50 knockdown (7- to 10-fold) (Table 1). PIP39 and TbTim50 are similar types of protein phosphatases (34, 35); however, there is no known connection between these two proteins that has been previously reported. Furthermore, PIP39 is localized primarily in glycosomes (35), whereas TbTim50 is localized in mitochondria (34). Therefore, a connection between TbTim50 and PIP39 appears significant. We also noticed that besides PIP39, a number of other glycosomal proteins were upregulated (1.8- to 2.0-fold). These include glycerol kinase, hexokinase, triose phosphate isomerase, ribokinase, phosphoenol pyruvate carboxy kinase, and fumarate reductase (Table 1). Glutaredoxin, which is a redox-regulatory protein in the mitochondrial IMS and cytosol (39), was upregulated about 2-fold in TbTim50 knockdown cells (Table 1). Among the downregulated proteins, we found a number of chaperone proteins, like Hsp70 (both mitochondrial and cytosolic), DNAj, glucose-regulated protein 78 (GRP78), cyclophilin, and calreticulin (Table 2 and Data Set S1). Several ribosomal proteins (RPs), such as 60S acidic protein, LP2, and S17, and the translation elongation factor EF2 were also downregulated due to TbTim50 knockdown (Table 2).

**TABLE 1:** Proteins upregulated due to TbTim50 knockdown^*a*^

Gene ID^*b*^	Description^*b*^^,^^*c*^	Avg KD/WT ratio^*d*^	*P* value^*e*^	Location(s)^*b*^^,^^*c*^
Tb927.9.6090	PIP39	10	0.002	Glyco
Tb927.6.3350	Hypothetical	3	0.02	Mito
Tb927.11.10020	Short-chain dehydrogenase	2.6	0.05	ND
Tb927.1.1770	Glutaredoxin	2.0	0.05	Mito/Cyto
Tb927.10.4230	NADH-Q reductase B13	2.0	0.05	Mito
Tb927.8.7170	Ionositol-1P-phosphatase	2.2	0.04	ND
Tb927.7.580	Deoxyribose-P aldolase	1.9	0.007	ND
Tb927.9.12570	Glycerol kinase	1.9	0.03	Glyco
Tb927.10.2010	Hexokinase	1.86	0.05	Glyco
Tb927.11.5520	Triose-P-isomerase	1.83	0.05	Glyco
Tb927.11.1020	Ribokinase	1.9	0.02	Glyco
Tb927.9.2320	POMP1	1.83	0.02	Mito
Tb927.8.4930	Hypothetical	1.75	0.05	Mito
Tb927.2.4210	PEPCK	1.76	0.03	Glyco
Tb927.10.3650	Fumarate reductase	1.6	0.03	Mito/Glyco

a Equal amounts of proteins of the crude mitochondrial lysates from parental control and TbTim50 knockdown T. brucei cells were digested with trypsin and analyzed by nano-LC-MS/MS. Mass spectra were searched against the NCBI database, and statistical analysis was performed from 2 biological replicates as described in Materials and Methods. Proteins that were upregulated >1.6-fold in the TbTim50 knockdown sample in comparison to the parental wild-type cells are shown. A complete list of the identified proteins is included in Data Set S1 in the supplemental material. PEPCK, phosphoenol pyruvate carboxykinase; Mito, mitochondria; Cyto, cytosol; Glyco, glycosome; ND, not determined.

b See www.tritrypdb.org.

c See www.ncbi.nlm.nih.gov/pubmed.

d Average ratio of spectral counts for peptides from wild-type (WT) cells to spectral counts for peptides from TbTim50 knockdown (KD) cells.

e Statistical significance was calculated from 2 biological replicates using Student’s *t* test.

**TABLE 2:** Proteins downregulated due to TbTim50 knockdown^*a*^

Gene ID^*b*^	Description^*b*^^,^^*c*^	Avg KD/WT ratio^*d*^	*P* value^*e*^	Location(s)^*b*^^,^^*c*^
Tb927.9.4500	Heat shock protein-like	0.5	0.03	
Tb927.9.7470	Nucleoside transporter	0.5	0.03	
Tb927.9.6130	Calmodulin	0.5	0.03	Cyto
Tb927.7.6890	Flagellar component	0.44	0.02	Flagella
Tb927.3.2310	Dynein heavy chain	0.44	0.22	Flagella
Tb927.2.5270	Cyclophilin	0.43	0.003	Cyto
Tb927.7.4770	Glycine dehydrogenase	0.42	0.03	Mito
Tb927.7.1910	Microtubule associated	0.42	0.04	Cyto
Tb927.9.2520	DEAD box helicase	0.42	0.009	Cyto
Tb927.11.2050	60S ribosomal protein	0.41	0.02	Cyto
Tb927.8.1330	60S ribosomal protein L7	0.41	0.03	Cyto
Tb927.9.11770	Adenylate cyclase	0.4	0.04	Mem
Tb927.8.6960	Fumarate hydratase	0.4	0.04	Mito
Tb927.3.4500	NADH dehydrogenase	0.4	0.015	Mito
Tb927.7.6360	Glucose regulatory protein 78	0.37	0.009	Cyto
Tb927.11.12880	POMP17	0.33	0.005	Mito
Tb927.11.5440	Malic enzyme	0.3	0.02	Mito
Tb927.9.11000	Small GTPase	0.29	0.03	Cyto
Tb927.11.11460	ATOM69	0.29	0.03	Mito
Tb927.7.710	Hsp70	0.29	0.02	Cyto/Mito
Tb927.11.6300	40S RP S5	0.29	0.02	Cyto
Tb927.11.11820	40S RP S17	0.25	0.03	Cyto
Tb927.11.1900	T complex	0.25	0.05	Cyto
Tb927.9.3370	Thioredoxin	0.25	0.05	Cyto/Mito
Tb927.10.2650	Elongation factor	0.22	0.003	Cyto
Tb927.10.2190	mEF	0.18	0.005	Mito
Tb927.10.9820	MIP	0.14	0.02	Mito

a Equal amounts of proteins of the crude mitochondrial lysates from parental control and TbTim50 knockdown T. brucei cells were digested with trypsin and analyzed by nano-LC-MS/MS analysis. Mass spectra were searched against the NCBI database, and statistical analysis was performed from 2 biological replicates as described in Materials and Methods. Proteins that were downregulated <0.6-fold in the TbTim50 knockdown sample in comparison to the parental wild-type cells are shown. A complete list of the identified proteins is included in Data Set S1 in the supplemental material. Mito, mitochondria; Cyto, cytosol; Mem, cell membrane; mEF, mitochondrial elongation factor; MIP, mitochondrial intermediate peptidase.

b See www.tritrypdb.org.

c See www.ncbi.nlm.nih.gov/pubmed.

d Average ratio of spectral counts for peptides from wild-type (WT) cells to spectral counts for peptides from TbTim50 knockdown (KD) cells.

e Statistical significance was calculated from 2 biological replicates using Student’s *t* test.

10.1128/mSphere.00353-19.8 DATA SET S1 Proteins up- and downregulated due to TbTim50 knockdown. Equal amounts of proteins of the crude mitochondrial lysates from parental control and TbTim50 knockdown T. brucei cells were digested with trypsin and analyzed by nano-LC-MS/MS. Mass spectra were searched against the NCBI database, and statistical analyses were performed from 2 biological replicates as described in Materials and Methods. Up- and downregulated proteins are highlighted in green and blue, respectively. Download Data Set S1, XLSX file, 0.3 MB. Copyright © 2019 Tripathi et al. 2019 Tripathi et al. This content is distributed under the terms of the Creative Commons Attribution 4.0 International license.

Next, we analyzed the proteins with significantly altered expression in Tim50 knockdown T. brucei cells using the STRING database (40) to identify any potential interactions among these proteins. The analysis was performed using T. brucei as the reference genome. All the different types of evidence were derived from either of four of the following sources: genomic context, high-throughput experiments, coexpression, and data mining (PubMed text mining). We noticed that the connected proteins would be classified into 4 to 5 clusters (Fig. S1). Cluster I includes ribosomal proteins and elongation factors, which were downregulated in T. brucei TbTim50 knockdown cells. Cluster II consists of several upregulated glycosomal proteins, and cluster III includes different chaperone proteins downregulated due to depletion of TbTim50. Three redox-regulated proteins were clustered into group IV. Among these, glutaredoxin was upregulated, whereas tryparedoxin peroxidase and thioredoxin were downregulated, due to TbTim50 knockdown. However, using these bioinformatics analyses, we could not find any previously established connection between PIP39 and TbTim50.

10.1128/mSphere.00353-19.1 FIG S1 Interaction map of the proteins that were significantly up- and downregulated due to TbTim50 knockdown. Protein enrichment analysis was performed using the STRING database as described in Materials and Methods. Based on the interaction patterns, proteins are classified into 4 groups. TPI, triose phosphate isomerase; HK, hexokinase; PEPCK, phosphoenol pyruvate carboxykinase; GD, glycine dehydrogenase; Rald, deoxyribose-phosphate aldolase; RK, ribose kinase; GK, glycerol kinase; GAPDH, glyceraldehyde-3-phosphate dehydrogenase; BCD, branched-chain α-ketoacid dehydrogenase; FR, fumarate reductase; FH, fumarate hydratase; AAT, alanine aminotransferase; ME, malic enzyme; Glu, glucosidase; Cal, calreticulin; Cyp, cyclophilin; GRP78, glucose-regulated protein 78; EF2, translation elongation factor 2; L7a, large ribosomal subunit protein 7a; S17, small ribosomal subunit protein 17; mEFG, mitochondrial translation elongation factor G; sG, small GTPase; T-com, T complex; TryPX, tryparedoxin peroxidase; Trdx, thioredoxin; GRX, glutaredoxin. Download FIG S1, TIF file, 0.5 MB. Copyright © 2019 Tripathi et al. 2019 Tripathi et al. This content is distributed under the terms of the Creative Commons Attribution 4.0 International license.

We also performed MudPIT analysis with isobaric tagging for relative protein quantitation (iTRAQ). T. brucei proteins from parental and TbTim50 knockdown cells were precipitated, digested, labeled with iTRAQ reagents, and analyzed by LC-MS/MS using a Thermo Q Exactive mass spectrometer. Mass spectra were searched against the combined UniProt KB/Swiss-Prot database (T. brucei and Homo sapiens). After applying protein filters (false discovery rate of <1% and at least two unique peptides), 3,090 proteins were identified in the two samples (Data Set S2). Among the proteins significantly affected, 122 proteins were statistically significantly upregulated, and 61 proteins were downregulated, in TbTim50 knockdown compared to wild-type cells (Data Set S2). These up- and downregulated proteins were then classified on the basis of their characterized and/or predicted functions and are presented in pie charts (Fig. S2). Major changes were noticed in the metabolic enzymes. We noted a slight upregulation of trypanosome alternative oxidase (TAO) and downregulation of several subunits of cytochrome oxidase (Data Set S2). We also noticed alteration in the levels of cytosolic and mitochondrial superoxide dismutases (SODs) (Data Set S2). When we overlap the data sets obtained from the label-free and iTRAQ proteomic experiments, we again found PIP39 to be the most affected due to the knockdown of TbTim50. Together, these results suggest that TbTim50 is connected with PIP39 by some unknown mechanism.

10.1128/mSphere.00353-19.2 FIG S2 Pie charts representing relative abundances of different groups of proteins that were down- and upregulated due to TbTim50 knockdown. Proteins were classified according to their gene ontology term and characterized functions. Download FIG S2, TIF file, 2.6 MB. Copyright © 2019 Tripathi et al. 2019 Tripathi et al. This content is distributed under the terms of the Creative Commons Attribution 4.0 International license.

10.1128/mSphere.00353-19.9 DATA SET S2 Effect of TbTim50 knockdown on T. brucei proteomes assessed by isobaric tagging for relative protein quantitation (iTRAQ) analysis. T. brucei proteins from parental and TbTim50 knockdown cells were precipitated, digested, labeled with iTRAQ reagents, and analyzed by LC-MS/MS. Mass spectra were searched against the UniProt KB/Swiss-Prot database. Statistical analyses were performed as described in Materials and Methods. Upregulated (>1.5 fold) and downregulated (<0.6-fold) proteins are highlighted in green and red, respectively. Download Data Set S2, XLSX file, 1.6 MB. Copyright © 2019 Tripathi et al. 2019 Tripathi et al. This content is distributed under the terms of the Creative Commons Attribution 4.0 International license.

### Validation of the proteomics results by immunoblot analysis.

**FIG 1:**
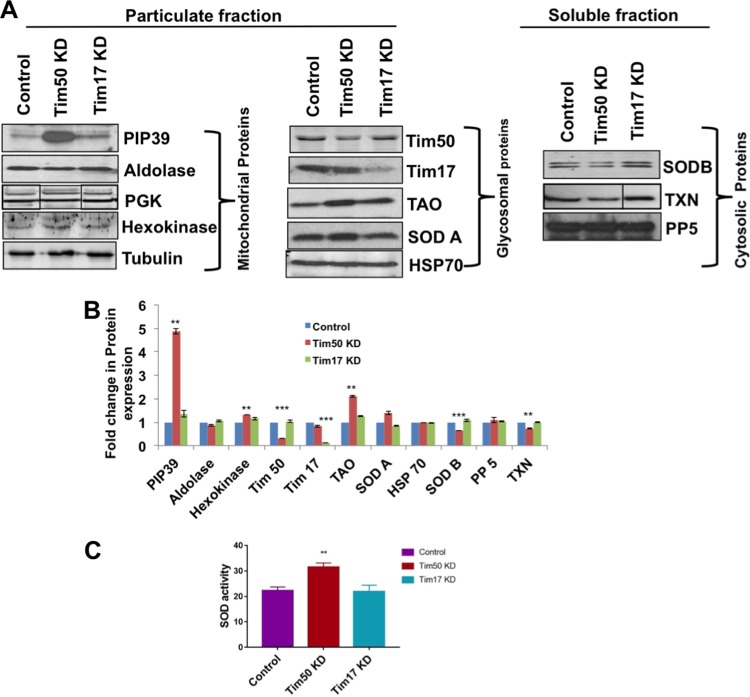
TbTim50 knockdown increases the levels of PIP39. (A) TbTim50 and TbTim17 RNAi cells were grown for 4 days in the presence of doxycycline. Wild-type control cells were also grown in parallel. Cells were extracted with 0.03% digitonin, and equal amounts of proteins from the particulate and cytosolic fractions were analyzed by immunoblotting using antibodies against PIP39, aldolase, PGK, and hexokinase as glycosomal proteins; TbTim17, TbTim50, TAO, SODA, and Hsp70 as mitochondrial proteins; and SODB, tryparedoxin peroxidase (TXN), and TbPP5 as cytosolic proteins. Tubulin and TbPP5 were used as the loading controls for the particulate and cytosolic fractions, respectively. Gels were spliced to remove unnecessary lanes. (B) Intensities of each protein band were quantitated by densitometric analysis and normalized with the intensities of the respective control protein bands. Relative levels of these proteins in TbTim50 KD and TbTim17 KD cells in comparison to the wild-type control were plotted. Data represent means ± standard errors from three experiments (*n* = 3). Values that are significantly different by a *t* test are indicated by bars and asterisks (**, *P* < 0.01; ***, *P* < 0.001). (C) Control, TbTim50 knockdown (Tim50 KD), and TbTim17 knockdown (Tim17 KD) T. brucei cells were solubilized in buffer as described in Materials and Methods, and SOD activity was measured. Standard errors were calculated from triplicates. Significance values were calculated by a *t* test and are indicated by asterisks (**, *P* < 0.01).

In order to verify our proteomic results, we performed immunoblot analysis for several T. brucei proteins. Similar to the proteomics results, we observed a large increase (∼5-fold) in the level of PIP39 by immunoblot analysis using specific antibodies in TbTim50 knockdown T. brucei cells in comparison to the parental control (Fig. 1A and B). However, PIP39 levels were unaltered when TbTim17 levels were reduced, suggesting that the observed effect on PIP39 levels is specific for TbTim50. We checked the levels of a few known glycosomal proteins, such as aldolase, phosphoglycerate kinase (PGK), and hexokinase. The aldolase level was slightly reduced in TbTim50 knockdown cells in comparison to control cells. However, hexokinase levels were slightly increased due to depletion of TbTim50, which was also correlated with our proteomics results. In support of these data, we also noticed that cell growth was inhibited more in low-glucose than in normal medium when TbTim50 levels were reduced by RNA interference (RNAi) (Fig. S3). We did not observe any significant changes in the levels of either of the two isoforms of PGK in the procyclic form. For mitochondrial proteins, we checked the levels of TbTim50, TbTim17, TAO, SODA, and Hsp70. The TAO level was upregulated more than 2-fold due to knockdown of TbTim50 (Fig. 1A and B). We previously reported that TbTim50 knockdown decreased the mitochondrial membrane potential and increased the levels of cellular reactive oxygen species (38). TAO is known to play role in reducing oxidative stress (41, 42). Therefore, increased levels of TAO may help TbTim50 knockdown cells to tolerate ROS. In addition, upregulation of TAO and hexokinase suggested a shift of energy production toward the glycolytic mode due to knockdown of TbTim50. We tested the levels of both the mitochondrial and cytosolic SODs SODA and SODB, respectively (43), using appropriate antibodies. The SODA level was upregulated about 1.5-fold, and the SODB level was reduced by half in comparison to the control (Fig. 1A and B). Furthermore, no significant changes of either SODA or SODB were observed in TbTim17 knockdown cells. We also measured SOD activity to verify our immunoblot results. We found that SOD activity was about 1.5-fold higher in TbTim50 knockdown than in parental control T. brucei cells, but TbTim17 knockdown cells did not show any change (Fig. 1C). The level of another protein phosphatase, PP5, which is localized in the cytosol (44), was not changed in any significant manner in these cells. However, cytosolic SODB and tryparedoxin levels were slightly reduced, and this was correlated with our proteomics results.

10.1128/mSphere.00353-19.3 FIG S3 Growth kinetics of TbTim50 RNAi cells in normal and low-glucose medium. TbTim50 RNAi cells were inoculated at a cell density of 3 × 10^6^ cells/ml in 5 mM glucose (+Gl) and low-glucose (≥5 μM) (−GL) medium in the presence (induced) and absence (uninduced) of doxycycline. Cell numbers were counted each day for 10 days postinduction. Cells were reinoculated when the parasite number reached 1 × 10^7^ cells/ml. The log cumulative cell number was plotted against days postinduction. Download FIG S3, TIF file, 2.3 MB. Copyright © 2019 Tripathi et al. 2019 Tripathi et al. This content is distributed under the terms of the Creative Commons Attribution 4.0 International license.

### PIP39 knockdown and overexpression do not have any effects on the levels of TbTim50.

**FIG 2:**
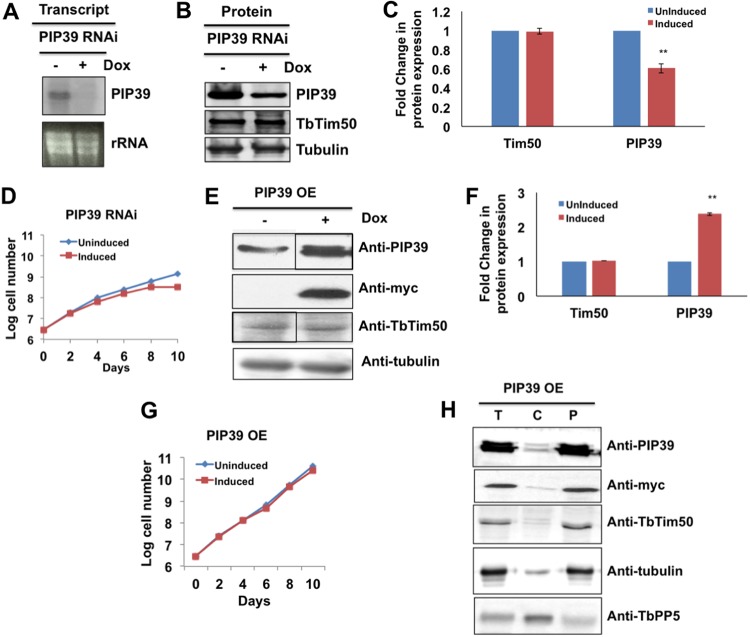
PIP39 knockdown and overexpression in the procyclic form of T. brucei. (A) Northern blot analysis of total RNAs isolated from PIP39 RNAi cells grown in the absence (−) and presence (+) of doxycycline (Dox) for 4 days. PIP39 cDNAs were used as the probe. Ethidium bromide-stained rRNA bands were used as the loading control. (B) Immunoblot analysis of total proteins harvested from PIP39 RNAi cells grown in the absence and presence of doxycycline for 4 days using PIP39 and TbTim50 antibodies. (C) Fold changes in the levels of TbTim50 and PIP39 in the induced cells were calculated in comparison to the uninduced control by densitometric quantitation and normalization with the corresponding tubulin bands from three experiments. (D) PIP39 RNAi cells were grown in the presence (induced) and absence (uninduced) of doxycycline. Cell numbers were counted each day for 10 days postinduction. Cells were reinoculated when the parasite number reached 1 × 10^7^ cells/ml. The log cumulative cell number was plotted against days postinduction. Standard errors were calculated from 3 experiments. (E) Immunoblot analysis of total proteins harvested from PIP39-overexpressing (OE) cells grown in the absence and presence of doxycycline for 4 days using PIP39, myc, TbTim50, and tubulin antibodies. Gels were spliced to remove unnecessary lanes. (F) Fold changes in the levels of TbTim50 and PIP39 in the induced cells were calculated in comparison to the uninduced control by densitometric quantitation. Significance values in panels C and F were calculated by a *t* test and are indicated by asterisks (**, *P* < 0.01). (G) PIP39-2×myc (PIP39 OE) cells were grown in the presence (induced) and absence (uninduced) of doxycycline. Cell numbers were counted each day for 10 days postinduction. Cells were reinoculated when the parasite number reached 1 × 10^7^ cells/ml. The log cumulative cell number was plotted against days postinduction. Standard errors were calculated from 4 independent experiments. (H) Total (T), cytosolic (C), and particulate (P) fractions were collected after solubilization of the cell membrane with 0.03% digitonin as described in Materials and Methods. Equal amounts of proteins (20 μg) were loaded per lane and immunoblotted with anti-PIP39, anti-TbTim50, anti-myc, antitubulin, and anti-TbPP5 antibodies. TbPP5 was used as the cytosolic marker protein.

To investigate if PIP39 and TbTim50 levels are complementary to each other, we investigated the effect of knockdown and overexpression of PIP39 in the procyclic form. PIP39 RNAi reduced the levels of PIP39 transcripts below the detection limit (Fig. 2A) and reduced the levels of PIP39 protein by 40% within 4 days postinduction (Fig. 2B and C). At this time point, cell growth was minimally affected; however, longer induction of RNAi (8 to 10 days) showed a reduction in cell growth (Fig. 2D). We did not find any significant differences in the TbTim50 levels in PIP39 knockdown cells (Fig. 2B and C), showing that the TbTim50 level is not regulated by PIP39. To further verify this point, we also overexpressed PIP39 with a 2×myc tag at the C terminus in the procyclic form (Fig. 2E). We did not observe any effect on TbTim50 levels due to overexpression of PIP39 (Fig. 2E and F). PIP39 overexpression in the procyclic form did not show any significant effect on cell growth (Fig. 2G). Expressed PIP39-myc was found in the particulate fraction (Fig. 2H). Together, these results therefore showed that PIP39 upregulation is a downstream signaling effect due to TbTim50 knockdown.

### Knockdown of TbTim50 alters cellular energy status and AMPK phosphorylation.

**FIG 3:**
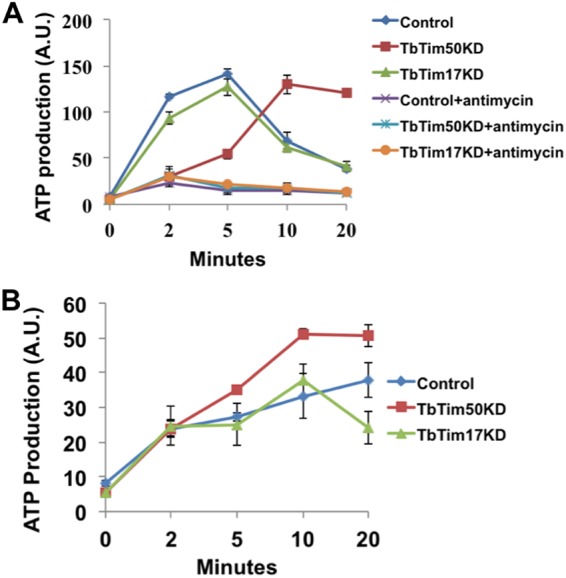
TbTim50 knockdown reduces the rate of oxidative phosphorylation but increases substrate-level phosphorylation in mitochondria. Mitochondria were isolated from wild-type control, TbTim50, and TbTim17 RNAi cells grown for 4 days in the presence of doxycycline. Equal amounts of mitochondrial proteins were incubated with the substrates succinate (A) and α-ketoglutarate (B). Succinate was added in duplicate samples in the absence and presence of antimycin. ATP production was started by the addition of ADP (55 μM). At different time points (0 to 20 min), samples were collected, proteins were precipitated by the addition of 60% perchloric acid, and the ATP concentration in the supernatants was measured by using a luciferase-based ATP assay kit as described in Materials and Methods. Relative light intensities in arbitrary units (A.U.) are plotted with time. Each experiment was done in triplicate.

Previously, we demonstrated that knockdown of TbTim50 reduced mitochondrial membrane potential to about 40% of the control within 4 days postinduction and that TbTim50 overexpression caused hyperpolarization of mitochondria (38). This indicates that TbTim50 is involved in maintaining the mitochondrial permeability barrier in T. brucei similarly to Tim50 in yeast and human. To investigate if depolarization of the mitochondrial inner membrane due to TbTim50 knockdown has any effect on ATP levels, we assessed the ATP production capacity of isolated mitochondria using succinate and α-ketoglutarate as the substrates, separately. ATP levels were increased sharply in control and TbTim17 knockdown mitochondria after the addition of succinate and reached maximum levels within 5 min (Fig. 3A). In contrast, the amount of ATP produced from succinate at the initial time point was very small in TbTim50 knockdown mitochondria; however, it reached levels similar to those of the control at later time points (Fig. 3A). The addition of antimycin A totally inhibited ATP production in all samples, as expected (Fig. 3A). On the other hand, when α-ketoglutarate was used as the substrate, ATP production by TbTim50 knockdown mitochondria sharply increased during the first 10 min and reached steady levels when the substrate had been depleted (Fig. 3B). At the 10-min time point, the level of ATP produced from TbTim50 knockdown mitochondria was about 1.7-fold higher, and at the 20-min time point, it was about 1.4-fold higher than in the wild-type control. The amount of ATP produced from TbTim17 knockdown mitochondria within 10 min was similar to that in the control; however, it dropped steadily at 20 min. Altogether, these results indicated that as a consequence of decreased mitochondrial membrane potential due to TbTim50 knockdown, oxidative phosphorylation decreased and substrate-level phosphorylation increased.

**FIG 4:**
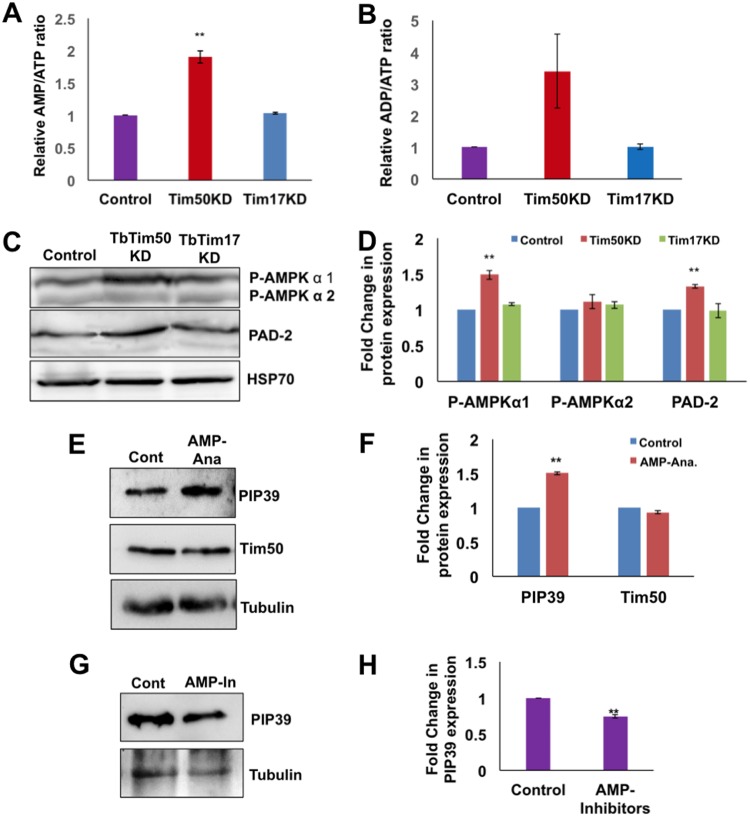
Effect of TbTim50 knockdown on cellular energy status and AMPK phosphorylation. TbTim50 and TbTim17 RNAi cells were grown for 4 days in the presence of doxycycline. Parental wild-type cells were also grown in parallel. Equal numbers of cells were harvested, washed with cold PBS, and lysed by perchloric acid. Cleared lysates were used to measure adenine nucleotide levels as described in Materials and Methods. (A and B) Relative ratios of AMP/ATP (A) and ADP/ATP (B) in TbTim50 and TbTim17 knockdown cells in comparison to the wild-type control. Standard errors were calculated from 3 independent experiments. (C) Equal amounts of total cell proteins from wild-type control and TbTim50 knockdown T. brucei cells were analyzed by immunoblot analysis using antibodies against phospho-AMPK, PAD array, and Hsp70. (D) Intensities of the P-AMPK-α1, P-AMPK-α2, and PAD2 bands were quantitated by densitometric analysis and normalized with the intensities of the corresponding Hsp70 protein bands. Relative levels of these proteins in TbTim50 and TbTim17 knockdown cells in comparison to the wild-type control are plotted. Averages and standard errors were calculated from three experiments. (E) Parental T. brucei 29-13 procyclic cells were incubated in the absence (control) and presence of 8-pCPT-2′-*O*-Me-cAMP-AM (AMP-Ana) for 24 h under normal culture conditions. Equal amounts of proteins from control and treated cells were analyzed by immunoblotting using PIP39, TbTim50, and tubulin antibodies. (F) Intensities of the PIP39 and TbTim50 bands were quantitated by densitometric analysis and normalized with the intensities of the corresponding tubulin protein bands. Relative levels of these proteins in treated cells in comparison to untreated control cells are plotted. Averages and standard errors were calculated from three experiments. (G) TbTim50 RNAi cells were induced with doxycycline in the absence (control) and presence of compound C (AMPK inhibitor [AMP-In]) as indicated in Materials and Methods. Equal amounts of cell proteins were analyzed by immunoblotting using PIP39 and tubulin antibodies. (H) Intensities of the PIP39 bands were quantitated, normalized with the corresponding tubulin band intensities, and plotted as a fold change in comparison to the untreated control. Standard errors were calculated from 3 independent experiments. Significance values in panels B, D, F, and H were calculated by a *t* test and are indicated by asterisks (**, *P* < 0.01).

To assess the effect of changes in the pattern of mitochondrial ATP production on cellular energy status, we estimated the ATP, ADP, and AMP levels in cell lysates from control, TbTim50 knockdown, and TbTim17 knockdown T. brucei cells. Cellular ATP levels were measured after the addition of both adenylate kinase (AK) and nucleoside diphosphate kinase (NDK), only NDK, and neither AK nor NDK to estimate the levels of AMP, ADP, and ATP in cells, respectively, as described in Materials and Methods. The results showed that cellular ATP levels decreased and AMP and ADP levels increased due to TbTim50 knockdown (Fig. 4A and B). The increases in the AMP/ATP and ADP/ATP ratios in Tim50 knockdown cells were ∼2-fold (Fig. 4A) and ∼3-fold (Fig. 4B), respectively, in comparison to the parental control. However, a similar effect was not observed in TbTim17 knockdown cells.

AMPK functions as a major sensor for cellular energy status and a master regulator of cell metabolism (45). AMPK is activated by AMP-dependent phosphorylation at Thr172 of the α-subunit of the enzyme (46). T. brucei possesses two isomeric forms of AMPK-α, AMPK-α1 and -α2. It has been shown that AMPK-α1 phosphorylation increases in response to an allosteric activator that mimics AMP and functions in metabolic regulation (46). The phosphorylation status of AMPK was assessed using a phosphospecific (T172-P) antibody (46). We found that TbTim50 knockdown increased the phosphorylation status of AMPK-α1 ∼1.6-fold in comparison to the wild-type control (Fig. 4C and D), which is correlated with the increased AMP levels in TbTim50 knockdown cells. The phospho-AMPK levels were minimally affected due to knockdown of TbTim17 (Fig. 4C and D). During differentiation of the long slender (LS) form to the stumpy bloodstream form, AMPK activation causes an upregulation of PAD1 expression (37); PAD1 is a marker for the latter form (47, 48). In the procyclic form, PAD1 (55 kDa) expression is decreased, with a concomitant upregulation of PAD2 (57 kDa), an isomeric form of PAD1. Using the PAD array antibody (47, 48), we observed a slight upregulation of the PAD2 level in the TbTim50 knockdown procyclic form (Fig. 4C and D). Hsp70 levels were unaltered in TbTim50 knockdown cells in comparison to the control. In order to investigate if AMPK activation in the procyclic form also has an effect on the expression levels of PIP39, we treated parental control cells with the cell-permeable AMP analog 8-(4-chlorophenylthio)-2′-*O*-methyladenosine-3′,5′-cyclic monophosphate (8-pCPT-2Me-cAMP). We found that PIP39 levels were increased about 1.5-fold after 24 h of treatment with 8-pCPT-2Me-cAMP (Fig. 4E and F), indicating that, similar to the bloodstream form (37), PIP39 upregulation is correlated with AMPK activation in the procyclic form. However, the levels of upregulation were relatively lower in the procyclic form, because this form already has a higher level of PIP39. In addition, we also treated TbTim50 RNAi cells with compound C, a known inhibitor of AMPK, simultaneously during induction of RNAi and found a decrease in the PIP39 levels in compound C-treated cells in comparison to untreated RNAi cells (Fig. 4G and H). Therefore, our results showed that AMPK activation due to a reduction in the levels of TbTim50 is at least partly responsible for upregulation of the PIP39 levels in the procyclic form.

### TbTim50 and TbPIP39 double-knockdown T. brucei cells become sensitive to oxidative stress.

**FIG 5:**
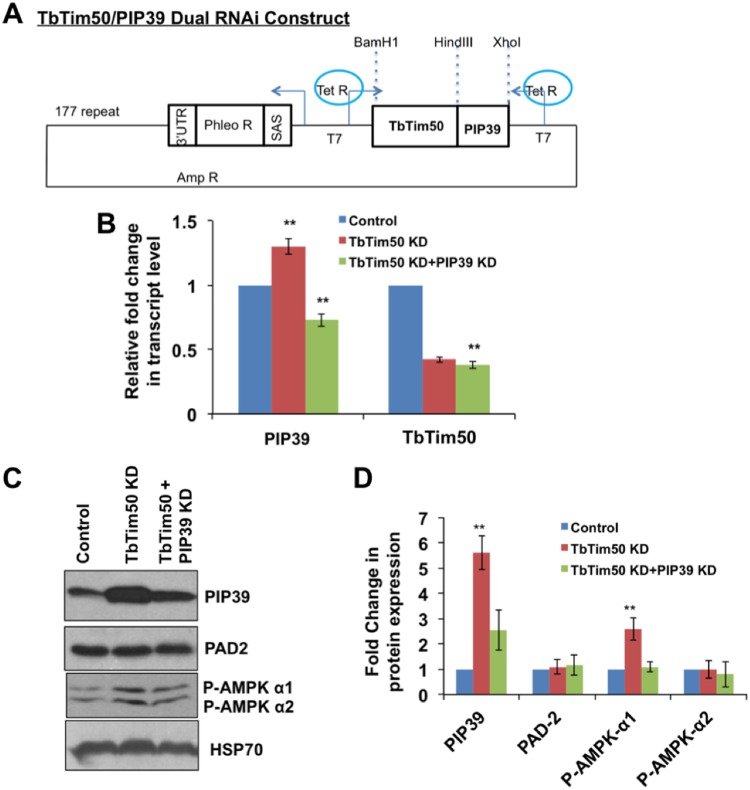
Double knockdown of TbTim50 and PIP39 and its effect on AMPK phosphorylation. (A) Schematic of the TbTim50 and PIP39 double-RNAi construct. Restriction sites for cloning the TbTim50 fragment and PIP39 are shown by dotted lines. Arrows indicate the direction of transcription of double-stranded RNA by the tetracycline-regulated T7 promoter and of the phleomycin resistance gene (Phleo R). The splice acceptor site (SAS) and 3′ untranslated region (UTR) for expression of the phleomycin resistance gene are shown. (B) Real-time PCR analysis of the TbTim50 and PIP39 transcripts in wild-type control, TbTim50 KD, and TbTim50 plus PIP39 KD T. brucei cells grown for 2 days in the presence of doxycycline. Telomerase reverse transcriptase was used as the internal control. Relative levels of the TbTim50 and PIP39 transcripts in comparison to the parental control were plotted. Averages and standard errors were calculated from three experiments. (C) Immunoblot analysis of the total cellular proteins from wild-type, TbTim50, and TbTim50 plus PIP39 RNAi cells after 4 days of induction with doxycycline using antibodies for PIP39, PAD array, and phospho-AMPK. (D) Intensities of each of the protein bands were quantitated by densitometric analysis and normalized with the intensities of the corresponding Hsp70 protein bands. Relative levels of these proteins in TbTim50 KD cells in comparison to the wild-type control are plotted. Averages and standard errors were calculated from three experiments. Significance values in panels B and D were calculated by a *t* test and are indicated by asterisks (**, *P* < 0.01).

From the correlation of PIP39 upregulation in TbTim50 knockdown cells and the increased tolerance of these cells to oxidative stress, we anticipated that PIP39 might be responsible for tolerance to exogenous oxidative stress in the procyclic form. To investigate this further, we doubly knocked down both proteins by expressing double-stranded RNAs for TbTim50 and PIP39 from the same vector (Fig. 5A) in the procyclic form. Real-time PCR analysis showed that TbTim50 RNAi reduced the transcript levels of TbTim50 to ∼0.4-fold. In the same cells, we found that the PIP39 transcript level was increased ∼1.3-fold. In double-knockdown cells, TbTim50 and PIP39 transcript levels were reduced to 0.4-fold and 0.65-fold, respectively (Fig. 5B). We did not observe any significant difference in cell growth due to double knockdown of TbTim50 and PIP39 (Fig. S4). Immunoblot analysis revealed that PIP39 levels were reduced in the particulate fraction more than 2-fold in double-knockdown cells in comparison to TbTim50 knockdown T. brucei cells, where it was upregulated about 5-fold compared to the control (Fig. 5C and D). Therefore, the PIP39 level was slightly higher in this cell fraction isolated from double-knockdown cells than in the wild-type control cells within 4 days postinduction. However, longer induction for 8 to 10 days showed that the levels of PIP39 were further reduced in double-knockdown procyclic cells (Fig. S5), showing that the PIP39 RNAi worked as expected in this cell line. However, in double-knockdown cells, PIP39 upregulation by TbTim50 knockdown and PIP39 downregulation by PIP39 RNAi happened simultaneously. As a result, the net effects of PIP39 levels were different in double- than in single-knockdown PIP39 RNAi cells. Interestingly, we found that AMPK phosphorylation was decreased in double-knockdown cells in comparison to TbTim50 knockdown T. brucei cells, indicating that AMPK activation is directly correlated with PIP39 levels in the procyclic form. The levels of PAD2 and Hsp70 were similar in all cell types (Fig. 5C and D).

10.1128/mSphere.00353-19.4 FIG S4 Effect of TbTim50 and PIP39 double knockdown on procyclic cell growth. TbTim50 and PIP39 double-RNAi cells were grown in the presence (induced) and absence (uninduced) of doxycycline. Cell numbers were counted each day for 10 days postinduction. Cells were reinoculated when the parasite number reached 1 × 10^7^ cells/ml. The log cumulative cell number was plotted against days postinduction. Download FIG S4, TIF file, 0.6 MB. Copyright © 2019 Tripathi et al. 2019 Tripathi et al. This content is distributed under the terms of the Creative Commons Attribution 4.0 International license.

10.1128/mSphere.00353-19.5 FIG S5 Levels of TbTim50 and PIP39 in single- and double-RNAi cells at different postinduction time points. (A) TbTim50 RNAi and TbTim50 plus PIP39 double-RNAi cells were grown in the presence of doxycycline. The parental control cells were also grown in parallel as controls. Cells were harvested at different postinduction time points, as indicated. Equal amounts of cell proteins were analyzed by immunoblotting using TbTim50, PIP39, and tubulin antibodies. (B) Band intensities for TbTim50 and PIP39 were quantitated by densitometry analysis and normalized with the corresponding tubulin band intensities, and average values from 3 independent experiments were plotted with calculated standard errors. Significance values were calculated by a *t* test and are indicated by asterisks (**, *P* < 0.01; ***, *P* < 0.001). Download FIG S5, TIF file, 1.8 MB. Copyright © 2019 Tripathi et al. 2019 Tripathi et al. This content is distributed under the terms of the Creative Commons Attribution 4.0 International license.

**FIG 6:**
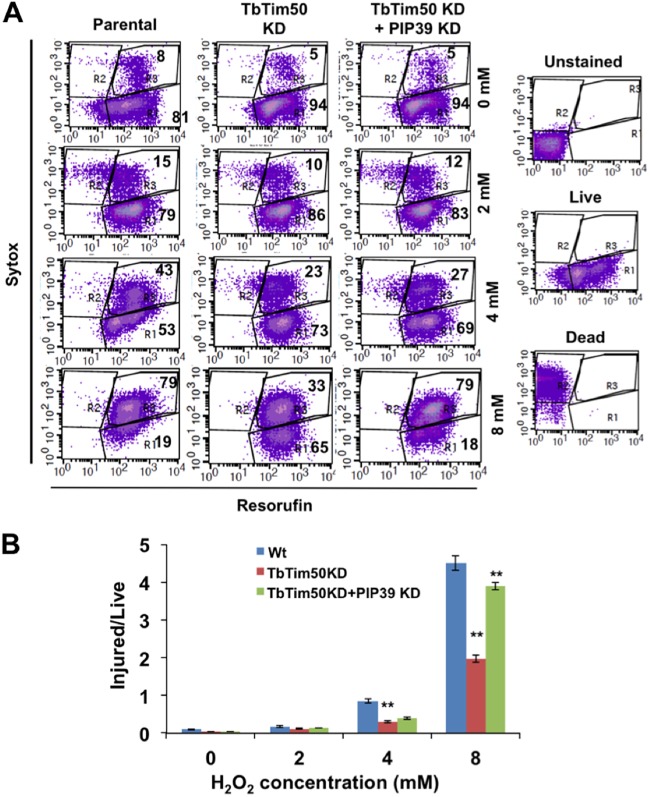
Effect of double knockdown of TbTim50 and PIP39 on T. brucei tolerance to oxidative stress. TbTim50 and TbTim50 plus PIP39 double-knockdown cells were grown for 4 days in the presence of doxycycline. Wild-type control cells were also grown in parallel. Equal numbers of cells were harvested and treated with different concentrations of H_2_O_2_ (0 to 8 mM) for 1 h. Cells were washed with PBS and stained with SYTOX green and resorufin as described in Materials and Methods. Fluorescence-activated cell sorter (FACS) analysis was performed immediately, and data were analyzed by density plots. (B) Ratios of injured to live cells (percent) calculated from three independent experiments and plotted versus the different cell types. Values shown are means ± standard errors of the means from triplicate samples. Significance values were calculated by a *t* test and are indicated by asterisks (**, *P* < 0.01). Wt, wild type.

To investigate the effect of oxidative stress on TbTim50 and PIP39 double-knockdown T. brucei cells, we incubated these cells in the presence of 0 to 8 mM H_2_O_2_ for 2 h and assessed cell injury by a live-dead assay as described in Materials and Methods. Our results clearly showed that TbTim50 knockdown cells were relatively more resistant to exogenously added H_2_O_2_, as we reported previously (38). Double knockdown of TbTim50 and PIP39 made T. brucei more sensitive to H_2_O_2_ than single TbTim50 knockdown cells, as indicated by the lower live-cell count as well as by the increased ratios of the percentages of injured/live cells (Fig. 6A and B). Measurement of the cellular ROS levels at 4 days postinduction showed a slight upregulation of ROS in double-knockdown (TbTim50 plus PIP39) cells in comparison to single knockdown for TbTim50 (Fig. S6A and B). However, single knockdown of PIP39 increased the levels of cellular ROS significantly (Fig. S6A and B), suggesting that higher levels of PIP39 may facilitate reduced cellular ROS in this form. Together, these results showed that increased phosphorylation of AMPK and upregulation of PIP39 rendered TbTim50 knockdown cells more tolerant to oxidative stress.

10.1128/mSphere.00353-19.6 FIG S6 Effect of single and double knockdowns of TbTim50 and PIP39 on cellular ROS levels. (A) TbTim50 and PIP39 single-knockdown and TbTim50 plus PIP39 double-knockdown cells were grown for 4 days in the presence of doxycycline. The parental control cells were also grown in parallel. Cells were harvested, stained with DCFH-DA as described in Materials and Methods, and analyzed by flow cytometry using emission and excitation wavelengths of 520 nm and 492 nm, respectively. Unstained cells and cells treated with H_2_O_2_ (8.0 mM) were used as negative and positive controls. FACS analysis results are shown in a histogram. (B) Fluorescence intensities were measured using FlowJo software and are plotted for different types of cells. Values for intensity are presented as arbitrary units. Standard errors were calculated from three independent experiments. Download FIG S6, TIF file, 1.8 MB. Copyright © 2019 Tripathi et al. 2019 Tripathi et al. This content is distributed under the terms of the Creative Commons Attribution 4.0 International license.

**FIG 7:**
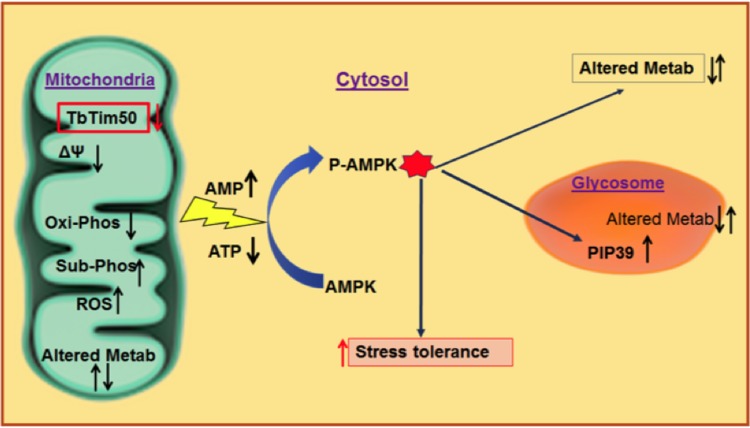
Schematic model for Tim50 function in stress tolerance in T. brucei. Changes in mitochondrial activities, cellular energy status (AMP/ATP ratio), levels of PIP39, and stress tolerance are indicated by upward and downward arrows. Phosphorylation of AMPK and cellular adaptation by altering metabolic activities in mitochondria, cytosol, and glycosomes due to TbTim50 knockdown are indicated.

Overall, we found that TbTim50 downregulation reduced mitochondrial membrane potential and ATP production that increased the cellular ROS level and the AMP/ATP ratio, which activated AMPK and upregulated PIP39 levels to protect cells from ROS, and cells became more tolerant to exogenous oxidative stress. A schematic for the role of TbTim50 and PIP39 in the maintenance of cellular homeostasis is shown in Fig. 7.

## DISCUSSION

In spite of a large amount of evidence showing that Tim50 plays additional roles besides mitochondrial protein import, the mechanism of its action for these cellular functions is not clearly understood in any system. Using quantitative proteomics analysis, here we identified a unique relationship between TbTim50 and a glycosomal protein, PIP39, which is also a CTD phosphatase like TbTim50, in T. brucei. We show that TbTim50 is required to maintain mitochondrial energy homeostasis and that TbTim50 cross talks with PIP39 via AMPK phosphorylation and dephosphorylation to maintain cellular functions. We also demonstrate that PIP39 is required for cellular tolerance to oxidative stress in the procyclic form of T. brucei.

Here, we demonstrate that TbTim50 downregulation caused an alteration in the cellular AMP/ATP ratio, and cells attempted to overcome this condition by altering their proteomes. The decrease in ATP levels in TbTim50 knockdown cells was due to a decreased rate of oxidative phosphorylation, which is most likely due to depolarization of the mitochondrial inner membrane. Mitochondria of the procyclic form of T. brucei produce ATP by both oxidative and substrate-level phosphorylation via acetate:succinate CoA transferase (ASCT cycle) (49). Reduction of electron flow via the oxidative pathway caused an increase in the level of TAO, as we observed. Upregulation of the levels of hexokinase also indicated that the TbTim50 knockdown cells depend more on glycolytic ATP production. Interestingly, we did not see a similar reduction in oxidative phosphorylation by knockdown of TbTim17, which is because the mitochondrial membrane potential was less affected in these cells (33, 34).

We have shown previously (38) and also in this paper that TbTim50 downregulation increased cellular ROS moderately, which is possibly due to a decreased rate of oxidative phosphorylation. The proteomics analysis and the follow-up validation experiments confirmed that TbTim50 downregulation increased the levels of PIP39 severalfold. In addition, we observed that PIP39 knockdown (single PIP39 RNAi) increased cellular ROS and decreased cell growth. However, overexpression of PIP39 was not harmful, indicating that PIP39 is required for cell fitness in the procyclic form. It is known that ROS acts as a second messenger that facilitates the communication between mitochondria and other cellular parts and that the amount of ROS determines the type of signal that it generates (50, 51). Multiple studies indicated that an elevation of mitochondrial ROS due to perturbed oxidative phosphorylation elicits an adaptive stress response phenomenon in cells (50). Therefore, together, all these results suggest that the higher level of PIP39 in TbTim50 knockdown cells could be an adaptive phenomenon to reduce cellular ROS. Although the mitochondrial stress response phenomenon is well studied in other organisms (51), this pathway has not been explored in T. brucei. However, it is known that T. brucei modulates its mitochondrial activities extensively to adapt under various stressful conditions during different life cycle stages, and several mitochondrial proteins have been found to be critical for stage differentiation (26, 52). Therefore, a dedicated communication pathway must exist between adaptation to mitochondrial dysfunction and alteration of gene expression, which in trypanosomes would be mostly posttranscriptional.

AMPK is critical for adaptive responses in human cells with mitochondrial dysfunction to oxidative stress (53). AMPK complexes have recently been functionally characterized in T. brucei (46). AMPK phosphorylation has also been found to be important for the transition from the LS to the stumpy bloodstream form (22, 23, 46) and for the connection between glycolysis and cell surface molecule expression in the procyclic form of T. brucei (54). Here, we show that mitochondrial dysfunction caused by TbTim50 downregulation increased the phosphorylation of AMPK and increased the levels of PIP39 in T. brucei. However, PIP39 up- or downregulation did not show any effect on the levels of TbTim50, which supports that PIP39 upregulation is a downstream signaling effect of TbTim50 knockdown. Furthermore, upregulation of PIP39 levels due to TbTim50 knockdown was perturbed by treatment of cells with an AMPK inhibitor, suggesting that, similarly to the bloodstream form, PIP39 expression is positively correlated with AMPK phosphorylation. We indeed found an upregulation of PIP39 by an AMP analog in the procyclic form.

PIP39 is an abundantly expressed protein phosphatase in the procyclic form; however, its function has not been fully explored in this form. During the transition from the stumpy bloodstream to the procyclic form, citrate and *cis*-aconitate are taken up by T. brucei by the cell surface transporters PAD1 and PAD2 and deactivate PTP1 (35, 37, 48). As a result, PIP39 is activated by phosphorylation. Activated PIP39 is transported to the glycosome, and this is necessary for differentiation and proliferation of T. brucei as the procyclic form (35). PIP39 is below detection levels in the replicating long slender (LS) bloodstream form but upregulated in the stumpy bloodstream form and is present at its highest levels in the procyclic forms (35, 37). Our studies show that PIP39 upregulation is required for adaptation of procyclic-form cells under oxidative stress, possibly via alteration of the metabolic pattern. ST bloodstream-form cells, where the PIP39 level is upregulated, are more tolerant to lower pH, exposure to protease, and lower temperatures (22, 23, 48) than the LS form, which has a very low level of PIP39. Furthermore, PIP39 levels and phosphorylation were increased when stumpy cells were treated at an acidic pH (48). Therefore, it can be assumed that PIP39 is somehow involved in cellular adaptive responses in the stumpy and procyclic forms. Overall, we found that depletion of TbTim50 caused mitochondrial dysfunction that triggers a signaling effect via AMPK activation. We found that AMPK activation and PIP39 upregulation are specific for TbTim50 depletion and not for TbTim17 knockdown that also causes mitochondrial dysfunction (33). Therefore, it could be a TbTim50-specific signaling event that requires further investigation.

Aspartate-based protein phosphatases that contain the DXDX(T/V) motif are primarily involved in dephosphorylation of Ser2 and -5 residues of the heptad repeat of the large subunit of the RNA polymerase II C-terminal domain to mediate transcriptional elongation and are known as CTD phosphatases (55). However, proteins belonging to this family also perform various specialized functions, like silencing of neuronal gene expression in nonneuronal tissues (56), bone morphogenesis (57), proteasome assembly (58), G_1_/S transition (59), and stress tolerance (60). T. brucei possesses a larger family of CTD phosphatase-like proteins. Apart from TbTim50 and PIP39, most of these proteins are uncharacterized in this organism. It would be interesting to see if the rest of the family members are also connected and what their functions are in T. brucei.

## MATERIALS AND METHODS

### Cells.

The procyclic form of T. brucei 427 29-13 expressing T7 polymerase and tetracycline repressor genes was grown in Schneider’s Drosophila medium-79 (SDM-79) medium containing 10% heat-inactivated fetal bovine serum in the presence of hygromycin (50 μg/ml) and G418 (15 μg/ml) (61) and was used as the parental control for our experiments. T. brucei cells expressing TbTim50 RNAi (TbTim50 KD) were previously developed (34). These cell lines were maintained in the same medium supplemented with phleomycin (2.5 μg/ml). Cell growth was assessed by inoculating the procyclic form at a cell density of 2 × 10^6^ cells/ml in fresh medium containing antibiotics in the presence or absence of doxycycline (1 μg/ml). Cells were counted at different growth time points with a Neubauer hemocytometer. The log cumulative cell number was plotted against incubation times.

### Generation of plasmid constructs and T. brucei transgenic cell lines.

The PIP39 RNAi constructs were generated by PCR amplifying the open reading frames (ORFs) of PIP39 using T. brucei genomic DNA as the template and sequence-specific primers (see Table S1 in the supplemental material). The forward and reverse primers were designed to add HindIII and BamHI restriction sites at the 5′ and 3′ ends of the ORFs, respectively. The PCR products were cloned into the tetracycline-inducible T7 double-headed-promoter p2T7^Ti^-177 RNAi vector between the HindIII and BamHI restriction sites. For the generation of the TbTim50 and PIP39 double-RNAi construct, a 505-bp fragment (residues 336 to 839) of the TbTim50 ORF was cloned into the HindIII and BamHI sites and the PIP39 ORF was cloned in tandem into the XhoI and HindIII sites of the p2T7^Ti^-177 RNAi vector. For overexpression of PIP39 with a 2×myc tag at the C terminus, the PIP39 ORF was cloned between the HindIII and XbaI sites of the pLew100-Myc vector. Plasmid DNAs were linearized by NotI digestion and transfected into T. brucei 29-13 cells. Transfected cells were selected with phleomycin (2.5 μg/ml).

10.1128/mSphere.00353-19.7 TABLE S1 Primers used in this study. The primers used for the generation of TbTim50 RNAi, PIP39 RNAi, and PIP39 overexpression and for quantitative reverse transcriptase PCR (TbTim50 qRT-PCR, PIP39 qRT-PCR, and telomerase reverse transcriptase qRT-PCR) are shown. Both forward and reverse primers are shown. All DNA sequences are in the 5′-to-3′ direction. Restriction enzyme sites are underlined. Download Table S1, DOCX file, 0.01 MB. Copyright © 2019 Tripathi et al. 2019 Tripathi et al. This content is distributed under the terms of the Creative Commons Attribution 4.0 International license.

### Multidimensional protein identification technology with label-free relative quantitation of protein levels.

Fresh mitochondrion pellets (50 μg proteins) were extracted in 20 μl radioimmunoprecipitation assay (RIPA) buffer (62) and immediately processed for trypsin digestion. Prior to LC-MS analysis, protein samples were denatured in 8 M urea and 50 mM Tris-HCl (pH 8.0), reduced with 10 mM Tris(2-carboxyethyl)phosphine hydrochloride (TCEP) for 60 min, alkylated with 2 mM iodoacetamide for 60 min, and then diluted in 2 M urea with 50 mM Tris-HCl (pH 8.0), at room temperature (RT). Two micrograms of trypsin gold (Promega) was added for overnight digestion (18 h at 37°C), and the tryptic peptides were then immediately desalted using Pierce C_18_ spin columns (Thermo Fischer Scientific) at RT. Peptides were eluted with 80% acetonitrile (ACN) and 0.1% formic acid (FA) and dried completely on a SpeedVac concentrator. For nano-LC-MS/MS analysis, Peptides were resuspended in 5 μl of 0.5% FA and loaded onto a 3-phase MudPIT column (150-μm by 2-cm C_18_ resin, 150-μm by 4-cm strong cation exchange [SCX] resin, filter union, and 100-μm by 12-cm C_18_ resin) as described previously (62). A 10-step MudPIT analysis (0 mM, 25 mM, 50 mM, 100 mM, 150 mM, 200 mM, 300 mM, 500 mM, 750 mM, and 1,000 mM ammonium acetate, with each salt pulse followed by a 120-min acetonitrile gradient of 5 to 50% buffer B [buffer A is 0.1% FA, and buffer B is 0.1% FA in acetonitrile]) was executed for LC-MS analysis using an Eksigent AS-1 autosampler and an Eksigent nano-LC Ultra 2D pump online with an Orbitrap LTQ XL linear ion trap mass spectrometer (Thermo Finnigan) with a nanospray source. MS data acquisition was done by a data-dependent 6-event method (a survey Fourier transform mass spectrometry [FTMS] scan [resolution of 30,000] followed by five data-dependent ion trap [IT] scans for the five consequent most abundant ions). The general mass spectrometric settings were as follows: spray voltage of 2.4 kV, no sheath and no auxiliary gas flow, ion transfer tube temperature of 200°C; collision-induced dissociation (CID) fragmentation (for MS/MS) with 35% normalized collision energy, activation *q* of 0.25, and activation time of 30 ms. The minimal threshold for the dependent scans was set to 1,000 counts, and a dynamic exclusion list was used with the following settings: repeat count of 1, repeat duration of 30 s, exclusion list size of 500, and exclusion duration of 90 s. For protein identification and quantification, database searches were done with PEAKS 8.5 software against the forward and reverse T. brucei and human trypsin sequences (downloaded from GenBank). The parameters for the database search were as follows: full tryptic digestion, up to 3 missed cleavage sites, 20 ppm for peptide mass tolerance, 0.5 Da for fragment mass tolerance, cysteine carbamidomethylation (+57 Da) as a fixed modification, and methionine oxidation (+16 Da) as a variable modification. Relative label-free quantification (LFQ) of the identified proteins was performed with the Q module of PEAKS software based on the extracted ion currents of the identified unique peptides’ parent ions or a spectral counting approach, and statistically significant changes were confirmed with Fisher’s exact test (*P* ≤ 0.005; Benjamini-Hochberg FDR of <0.05).

### iTRAQ mass spectrometry analysis.

Proteins in the cell lysate were precipitated with ice-cold acetone overnight at −20°C, washed with cold acetone, dried, and reconstituted in 8 M urea in 250 mM triethylammonium bicarbonate (TEAB) (pH 8.0). Samples were reduced with 8 μl of 50 mM TCEP, alkylated with 4 μl of 200 mM methylmethane thiosulfonate (MMTS), diluted with TEAB to obtain a final solution containing 2 M urea, and digested with sequencing-grade trypsin overnight. Peptides were labeled with iTRAQ reagents according to the manufacturer’s instructions (Sciex). The labeled peptides were then desalted by a modified stage tip method as described previously (63) and analyzed on a self-packed biphasic C_18_/SCX MudPIT column using a helium-pressurized cell (pressure bomb) and with a 13-step salt pulse gradient (0 mM, 25 mM, 50 mM, 75 mM, 100 mM, 150 mM, 200 mM, 250 mM, 300 mM, 500 mM, 750 mM, 1 M, and 2 M ammonium acetate). The peptides were eluted from the reverse-phase analytical column using a gradient of 2 to 98% ACN, followed by equilibration at 2% ACN. A Q Exactive mass spectrometer (Thermo Scientific), equipped with a nanoelectrospray ionization source, was used to mass analyze the eluting peptides. Peptide/protein identifications and quantitative analysis were performed using Spectrum Mill (Agilent) as described previously (63). MS/MS spectra were searched against a subset of the UniProt KB protein database containing T. brucei proteins. Autovalidation procedures in Spectrum Mill were used to filter the data to <1% false discovery rates at the protein and peptide levels. Log_2_ protein ratios were fit to a normal distribution using nonlinear (least-squares) regression, and the mean and standard deviation values derived from the Gaussian fit of the ratios were used to calculate *P* values. Subsequently, *P* values were corrected for multiple comparisons by the Benjamini-Hochberg method (64, 65). Corrected *P* values of <0.05 were considered significant.

### Bioinformatics analysis.

Pathway enrichment analysis was performed using DAVID Bioinformatics Resources 6.7, NIAID/NIH (66), and the STRING database (39) with the KEGG database (67) as a reference. The identified enriched pathways with *P* values of <0.05 were selected. Protein family enrichment was done using the STRING database with Pfam as the reference database. The Pfam database is a database of protein families, each represented by multiple-sequence alignments and hidden Markov models (HMMs) (68). Functional analysis of proteins was performed using InterPro to classify the proteins into families and to predict domains and important sites. Enrichment analysis of proteins with InterPro was performed with the STRING database.

### qRT-PCR analysis.

For quantitative real-time PCR (qRT-PCR) analysis, RNA was isolated from T. brucei cells using an RNeasy miniprep isolation kit (Qiagen) and digested with amplification-grade DNase (1 U/μl) (Invitrogen) for 1 h before first-strand cDNA synthesis using the iScript cDNA synthesis kit (Bio-Rad). The cDNA was made from 2 μg RNA using the iScript cDNA synthesis kit (Bio-Rad). Real-time quantitative PCR was performed with a CFX96 Touch real-time PCR detection system (Bio-Rad). The resulting cDNA was amplified using SsoAdvanced universal SYBR green supermix (Bio-Rad) and gene-specific primers, as indicated (Table S1).

### Digitonin extraction.

Procyclic cells (2 × 10^8^) were resuspended in 500 μl of SMEP buffer (250 mM sucrose, 20 mM morpholinepropanesulfonic acid [MOPS]-KOH [pH 7.4], 2 mM EDTA, 1 mM phenylmethylsulfonyl fluoride [PMSF]) containing 0.03% digitonin and incubated on ice for 5 min. The cell suspension was then centrifuged for 5 min at 6,800 × *g* at 4°C. The resultant pellet was considered the particulate fraction, and the supernatant contained soluble cytosolic proteins.

### Isolation of mitochondria.

Mitochondria were isolated from the parasite after lysis by nitrogen cavitation in isotonic buffer (33, 34). The isolated mitochondria were stored at a protein concentration of 10 mg/ml in SME buffer containing 50% glycerol at −70°C. Before use, mitochondria were washed twice with 9 volumes of SME buffer to remove glycerol.

### SDS-PAGE and immunoblot analysis.

Proteins from isolated mitochondria and the total cell extract were measured by using the Bradford reagent, and equal protein samples were resolved on an SDS-PAGE gel and then transferred to nitrocellulose membranes (Bio-Rad). The antigens were visualized by using the West Pico chemiluminescent substrate (Thermo Fisher). Antibodies against TbTim50 (34), SODA (46), SODB (46), PIP39 (35), TAO (69), Hsp70 (70), TbTim17 (33), TbPP5 (44), aldolase (71), PGK (72), hexokinase (73), and tryparedoxin peroxidase (TXN) (74) were used as probes. Phospho-AMPK-α (P-Thr172) antibody was purchased from Cell Signaling Technologies. The secondary antibodies used were either anti-rabbit or anti-mouse immunoglobulins linked to horseradish peroxidase (Thermo Fisher).

### SOD activity assay.

SOD activity was measured as described previously (75), with some modifications. Cells were centrifuged, resuspended in STE buffer (0.25 M sucrose, 25 mM Tris-HCl, 1 M EDTA [pH 7.8]), and disrupted by three cycles of sonication (30 s each at 60 W). The suspension was centrifuged (2,500 × *g* for 10 min at 4°C), and the supernatant was collected. Protein concentrations of the supernatant fractions were determined using the Bradford method (76), and equal amounts of proteins were used for an enzyme assay. The levels of SOD in lysates were measured in triplicate by using commercially available SOD kits (Sigma-Aldrich), according to the manufacturer’s instructions, in a 96-well plate in duplicate. The plate was incubated at 37°C for 20 min, and the absorbance at 450 nm was measured using a microplate reader (Bio-Rad).

### Measurement of levels of adenine nucleotides.

The ATP concentration was measured using an ATP bioluminescence assay kit (Invitrogen) as described previously (77). Cellular ATP, ADP, and AMP levels were measured as described previously (77–79). Cells (1 × 10^8^) were lysed by the addition of 300 μl of 0.5 M HClO_4_ on ice for 30 min. The clear lysates were neutralized (75). For measurement of ADP and AMP, 200 μM dCTP was added. Each sample was used in two separate reactions: one to measure ATP plus ADP plus AMP by adding adenylate kinase and nucleoside diphosphate kinase (NDK) and the other to measure ADP plus ATP by adding NDK only. The amount of ATP was measured by an ATP determination kit (Invitrogen) as described above. The levels of ADP were determined by subtracting the ATP values from the ADP plus ATP values, and the amount of AMP was calculated by subtracting the ADP plus ATP values from the AMP plus ADP plus ATP values.

### AMPK inhibition by compound C.

TbTim50 RNAi cells were induced with doxycycline (1 μg/ml) and incubated with 0.25 mM compound C for 24 h at 27°C. Cells were harvested and lysed with RIPA buffer, and equal amounts of proteins were used for immunoblot analysis.

### AMP analog treatment.

Procyclic Trypanosoma brucei *brucei* (29-13) cells (5 × 10^6^ cells/ml) were incubated in the presence and absence of 8-pCPT-2′-*O*-Me-cAMP-AM (10 μM) (Tocris Bioscience) under normal culture conditions for 24 h. Cells were harvested and lysed with RIPA buffer, and equal amounts of proteins were used for immunoblot analysis.

### H_2_O_2_ treatment.

TbTim50 KD and TbTim50 plus PIP39 double-KD T. brucei cells were induced for 4 days with doxycycline (1 μg/ml). Parental T. brucei wild-type cells were also grown in parallel for 4 days. Cells were then exposed to different concentrations of hydrogen peroxide (0 to 8 mM) for 1 h. Treated cells were immediately used for further analysis.

### Live-dead assay.

T. brucei parental 427 29-13, TbTim50 KD, and TbTim50 plus PIP39 KD cells (2 × 10^7^) were harvested, washed once with 1× phosphate-buffered saline (PBS), and resuspended in 200 μl of 1× PBS. C12-resazurin and SYTOX green dyes (Molecular Probes) were then added to each 100-μl cell suspension at a final concentration of 500 nM. Cells were incubated at 27°C for 15 min. Next, 400 μl of 1× PBS was added to the cells, and the mixtures were kept on ice. The samples were analyzed immediately by flow cytometry (BD FACSCalibur), with excitation at 488 nm and measurement of the fluorescence emission at 530 nm and 575 nm. Data were analyzed with CellQuest.

### Measurement of ROS levels.

T. brucei cells (1 × 10^7^ cells/ml) were washed with 1× PBS and incubated with 10 μM 2′,7′-dichlorohydrofluorescein diacetate (DCFH-DA) for 30 min at 27°C. After incubation, cells were washed once with 1× PBS and analyzed by flow cytometry, by measuring the fluorescence emission at 520 nm using the excitation wavelength at 492 nm.

### Densitometry and statistical analyses.

ImageJ software (NIH) was used to perform densitometry of Western blots. The band intensity of the loading control was used for normalization, and values were plotted using MS Excel. Standard errors were calculated form three independent experiments for each blot. The data were compared with the values for the control and analyzed for statistical significance by using the PRISM (GraphPad) *t* test. *P* values are indicated by asterisks in the figures.

### Data availability.

Mass spectrometry data have been deposited in TriTrypDB (kinetoplastid genomic resource, https://tritrypdb.org/tritrypdb/) under the gene IDs given in this paper.
