# Erratum for Tripathi et al., “The Cross Talk between TbTim50 and PIP39, Two Aspartate-Based Protein Phosphatases, Maintains Cellular Homeostasis in Trypanosoma brucei”

**DOI:** 10.1128/mSphere.00607-19

**Published:** 2019-09-04

**Authors:** Anuj Tripathi, Ujjal K. Singha, Victor Paromov, Salisha Hill, Siddharth Pratap, Kristie Rose, Minu Chaudhuri

**Affiliations:** a Department of Microbiology, Immunology, and Physiology, Meharry Medical College, Nashville, Tennessee, USA; b Department of Biochemistry, Vanderbilt University, Nashville, Tennessee, USA

## ERRATUM

Volume 4, no. 4, e00353-19, 2019, https://doi.org/10.1128/mSphere.00353-19. This article was published on 7 August 2019 with the fifth author’s name misspelled. The byline has been updated in the current version, posted on 30 August 2019.

