# Supplementary figures and images for: The Cross Talk between TbTim50 and PIP39, Two Aspartate-Based Protein Phosphatases, Maintains Cellular Homeostasis in Trypanosoma brucei
Source: mSphere. 2019 Aug 7;4(4):e00353-19. doi: 10.1128/mSphere.00353-19 (PMC6686227; doi:10.1128/mSphere.00353-19)

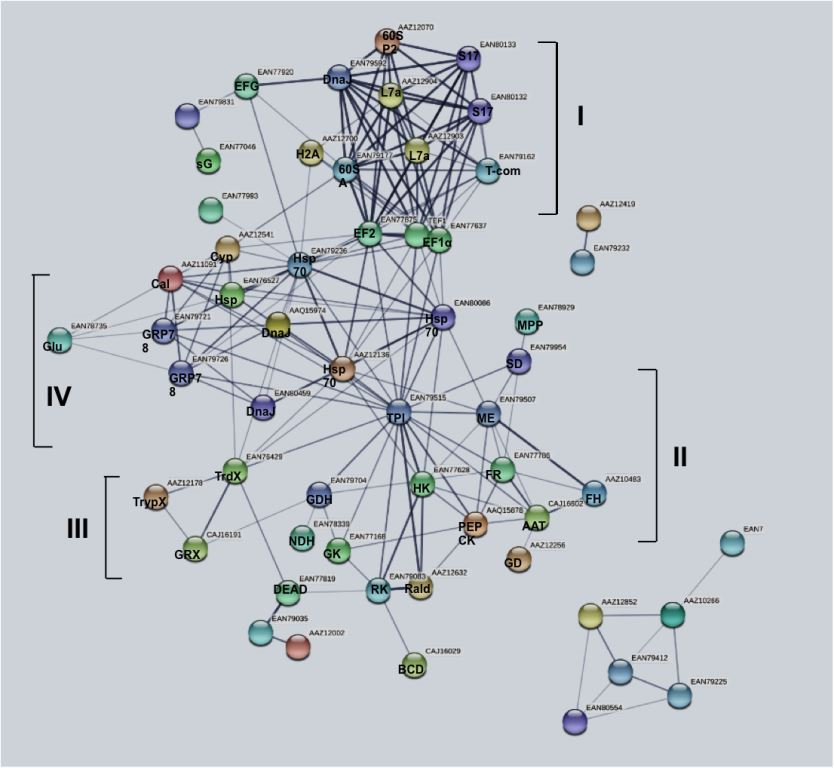

Supplement: FIG S1 [file mSphere.00353-19-sf001.tif]

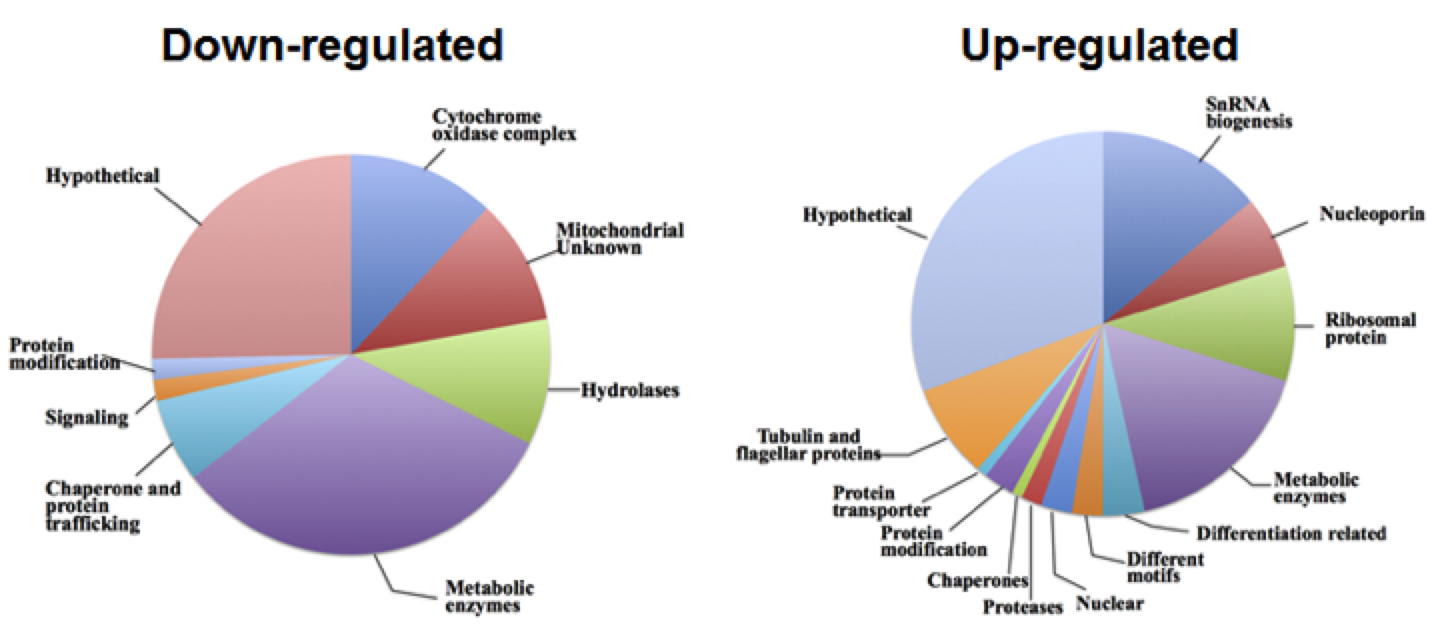

Supplement: FIG S2 [file mSphere.00353-19-sf002.tif]

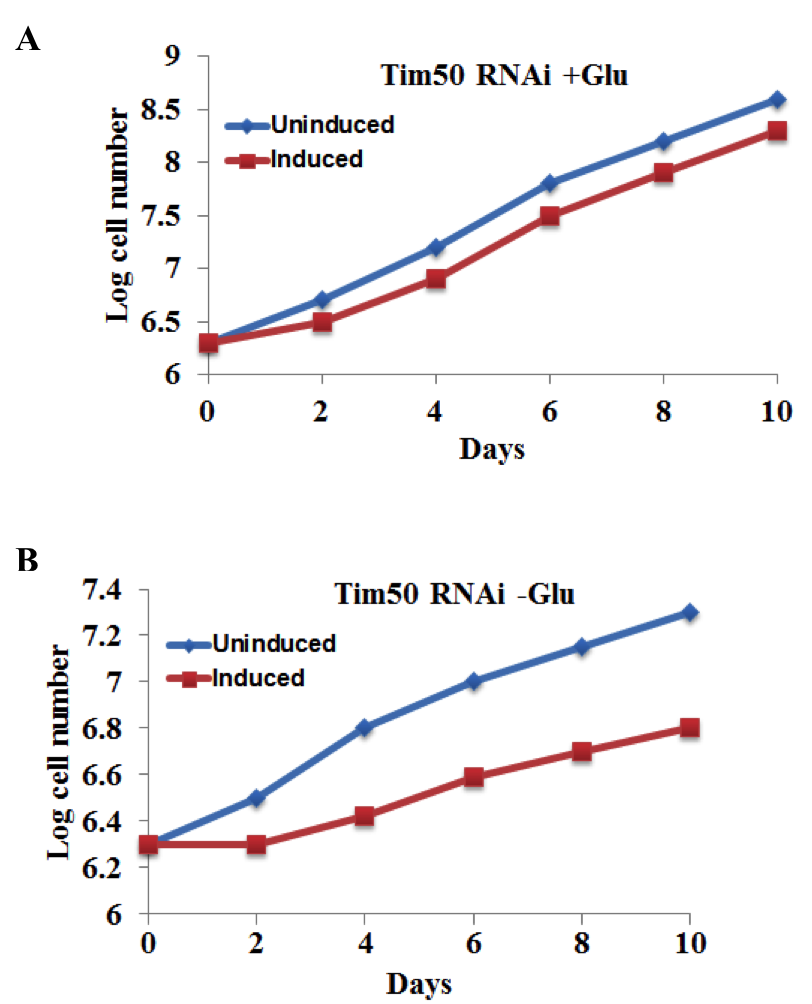

Supplement: FIG S3 [file mSphere.00353-19-sf003.tif]

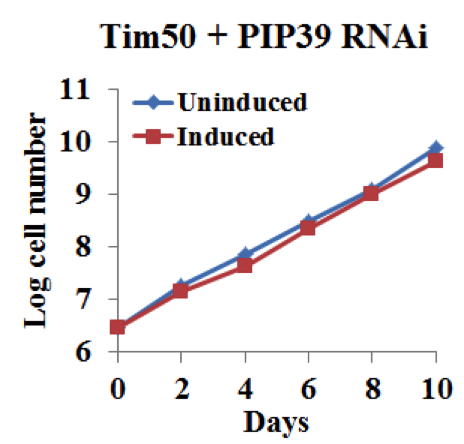

Supplement: FIG S4 [file mSphere.00353-19-sf004.tif]

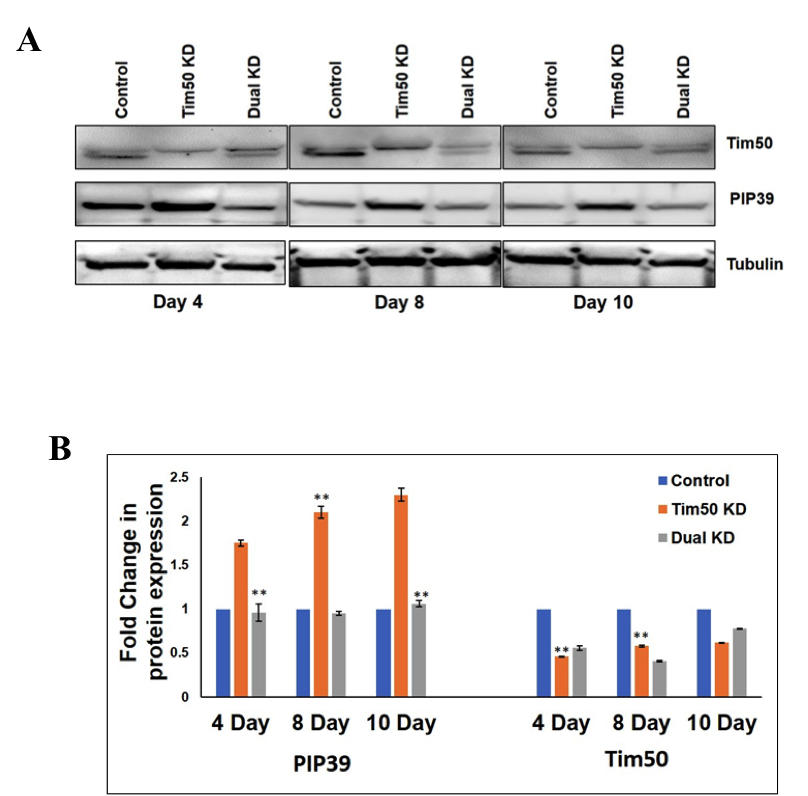

Supplement: FIG S5 [file mSphere.00353-19-sf005.tif]

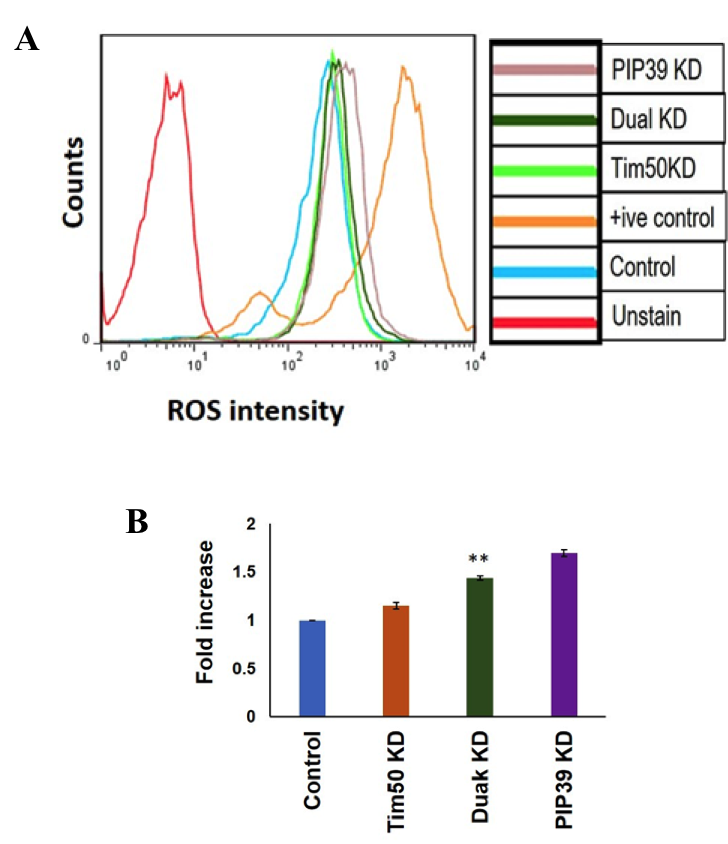

Supplement: FIG S6 [file mSphere.00353-19-sf006.tif]
