# Supplementary material for: The Cross Talk between TbTim50 and PIP39, Two Aspartate-Based Protein Phosphatases, Maintains Cellular Homeostasis in Trypanosoma brucei
Source: mSphere. 2019 Aug 7;4(4):e00353-19. doi: 10.1128/mSphere.00353-19 (PMC6686227; doi:10.1128/mSphere.00353-19)
Supplement: TABLE S1 [file mSphere.00353-19-st001.docx]

**Table S1. Primers used in this study**

Primer Name Direction Sequence* (5′-3′)

TbTim50 RNAi Forward AGTCGGATCCGCATAGAGGGG

AAAAGAGTGAGG

TbTim50 RNAi Reverse AGTCAAGCTTGGACGAAAAGCAA

CATAAACGGTG

PIP39 RNAi Forward GATCAAGCTTCGACACGCTTTT

CACGCAAC

PIP39 RNAi Reverse GATCGAGCTCTGACACGATTCAA

TGGTCTGCC

PIP39 OE Forward GATCAAGCTTATGGTGAGGACG

ACACGC

PIP39 OE Reverse GATCTCTAGAAAGTCTTGAAGG

AGTGTG

TbTim50qRT-PCR Forward CCGCCTCCGTCTCGGTTTAT

TbTim50qRT-PCR Reverse CCAGGTCCCGACCAAGCAAT

PIP39qRT-PCR Forward TGGAGGTGCAGGTGTTACAA

PIP39qRT-PCR Reverse CGTTTGAGTTCGGGAAAGGG

^1^TERTqRT-PCR Forward GAGCGTGTGACTTCCGAAGG

^1^TERTqRT-PCR Reverse AGGAACTGTCACGGAGTTTGC

*Restriction enzyme sites are underlined

^1^TERT; Telomerase reverse transcriptase was used as a reference gene for transcript quantification (Brenndorfer and Boshart 2010. Mol Biochem Parasitol 172;52-55)
